# Acroform type of enchondromatosis associated with severe vertebral involvement and facial dysmorphism in a boy with a new variant of enchondromatosis type I1 of Spranger: case report and a review of the literature

**DOI:** 10.1186/1757-1626-1-324

**Published:** 2008-11-18

**Authors:** Ali Al Kaissi, Katharina Roetzer, Klaus Klaushofer, Franz Grill

**Affiliations:** 1 Ludwig-Boltzmann Institute of Osteology at the Hanusch Hospital of WGKK and AUVA Trauma Centre Meidling, 4th Medical Department, Hanusch Hospital, Vienna, Austria; 2 Orthopaedic Hospital of Speising, Paediatric Department, Vienna, Austria; 3 Institute of Human Genetics, Medical University of Graz, A-8010 Graz, Austria

## Abstract

**Background:**

Enchondromatosis represent a heterogenous group of disorders. Spranger et al attempted a classification into 6 types: Ollier disease, Maffuci syndrome, metachondromatosis, spondyloenchondrodysplasia, enchondromatosis with irregular vertebral lesions, and generalized enchondromatosis. Halal and Azouz added 3 tentative categories to the 6 in the classification of Spranger et al.

**Case presentation:**

We report on a 15-year-old boy with acrofrom upper limbs and mixed appearance of radiolucency, cysts and striae of fibro-chondromatosis. Lower limbs (femoral, tibial and fibular dysplasia showed enlarged metaphyses near the knees bilaterally) were present. Additional features of short stature, macrocephaly, facial dysmorphism, and generalised platyspondyly have been encountered. These bone shortenings were associated with bone bending, curving and rhizomelia of the upper limbs with significant macrodactyly. Limitations in articular movements were present. The forearm deformities were similar to those observed in hereditary multiple exostosis.

**Conclusion:**

The acrofrom upper limbs with mixed appearances of radiolucencies, cysts and striae of fibro-chondromatosis are the basic features of type I1Spranger. The constellation of facial dysmorphic features and significant vertebral abnormalities in our present patient were not compatible with the above-mentioned type of enchondromatosis. Our report widens the knowledge of disorders characterised by enchondromatosis. Ascertainment of the mode of inheritance in our present patient was difficult because of insufficient family history and parents declined clinical/radiographic documentation.

## Background

Enchondromas are almost exclusively localized in the metaphysis of long bones and in the small bones of the hands and feet. They are initially localized close to the growth plate cartilage and then migrate progressively towards the diaphysis. The epiphyseal region next to an affected metaphysis may show irregularities. Enchondromas result in severe growth abnormalities (more severe than those observed in multiple exostosis). Affected diaphyses are short and massively enlarged, and these may show bending close to the metaphysis. Ulnar shortening is usually more relevant than shortening of the radius. Fingers often show irregular sizes. Signs of pathological fractures may be present. Ollier was the first to describe a syndrome of multiple enchondromatosis [1-4]. Ollier disease may be unilateral or bilateral. When bilateral it is always asymmetrical and, as with Maffucci syndrome and metachondromatosis, there is classically no vertebral involvement. Marked swellings are evident on both hands and feet [5]. We present details of a skeletal dysplasia in a15-year-old boy who manifested phenotypic features consistent but not absolutely compatible with the classic acrofrom type of multiple enchondromatosis of type I1 Spranger nor with the other types of enchondromatosis.

## Clinical report

The patient was referred at the age of 12 years with shortening of his right leg of 10 cm (3.4 cm tibia and 6.6 cm of the femur). In addition, there was a varus deformity at the level of the distal femur and a valgus deformity at the level of the proximal tibia. The boy was born to non- related parents at 39 weeks of uncomplicated gestation. His growth parameters at birth were around the 10 th percentile. His subsequent course of development was retarded and the child's performance was subnormal.

At early childhood he developed marked swellings of palpable bony masses. These bony masses were bilateral and symmetrical in distribution over the forearms and the small tubular bones of the hands and feet with severe deformity of the involved segments. Rhizomelic upper limb shortening associated with bone bending and curving. The hands showed Madelung's- like deformity and macrodactyly.

**Figure 1:**
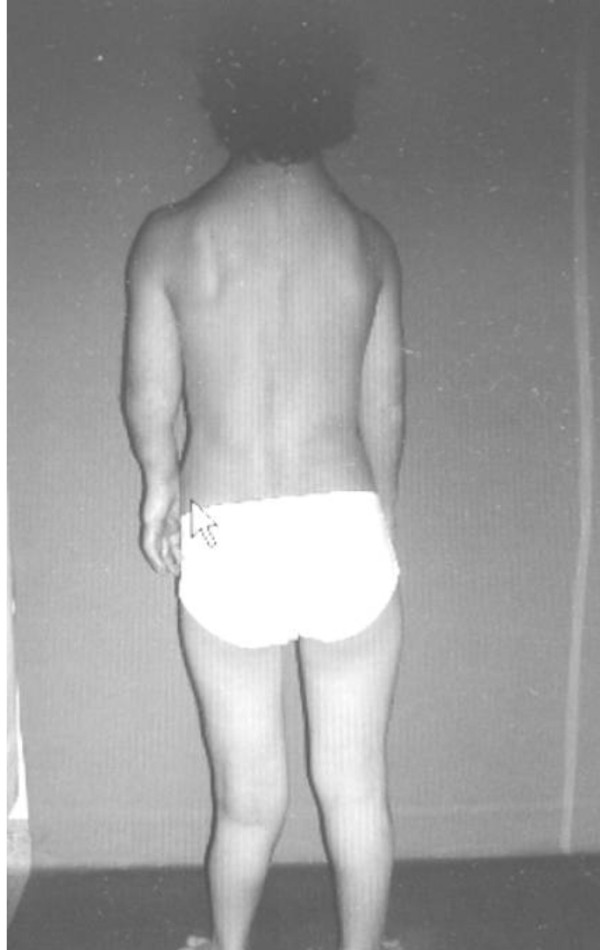
**Patient's photo showed macrocephaly, a course dysmorphic facies (frontal bossing, downslanting palpebral fissures, hypertelorism, long philtrum, broad and large nose and macrostomia).** There was generalized ligamentous hyperlaxity associated with genu valgum, valgus ankles and pes planus. Short stature and rhizomelia were evident.

**Figure 2:**
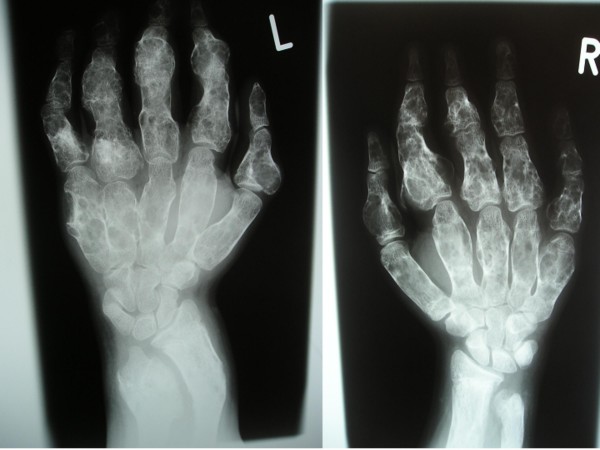
**The anteroposterior hands and forearms radiograph showed irregularly expanded metaphyses and shortened diaphyses were curved over the perimetaphyseal region.** Ovoid, cystic and highly radiolucent lesions, elongated parallel to the major axis of the bone, originating near the physis and migrating towards the diaphyses with growth. Shortenings, associated with bone bending causing effectively the development of Madelung's-like deformity.

**Figure 3:**
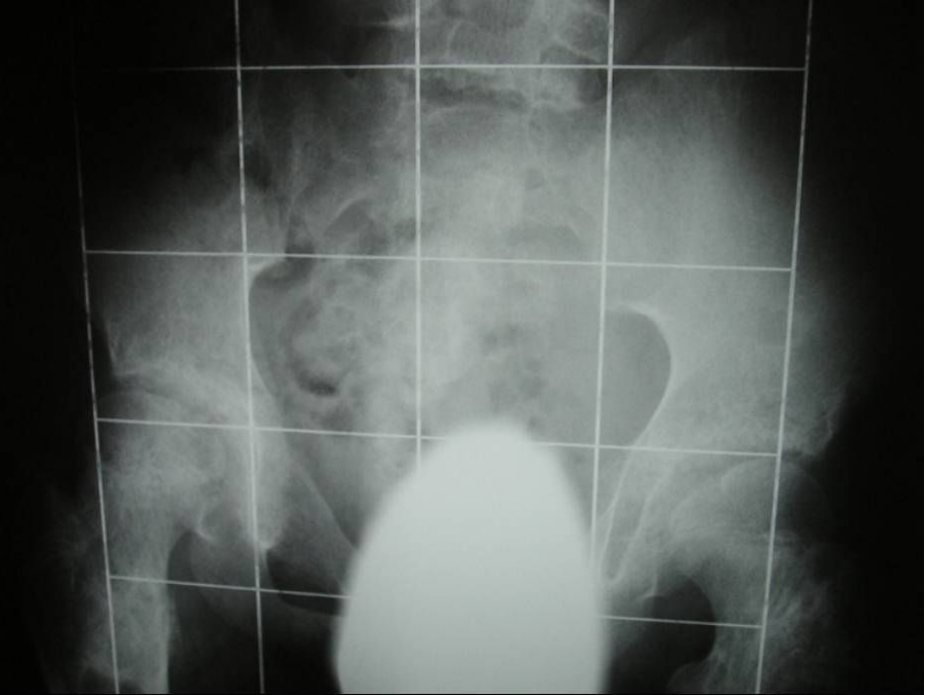
Anteroposterior pelvic radiograph showed coxa valga associated with defective modeling of the femoral necks and extensive striae of fibro-chondromatosis type.

**Figure 4:**
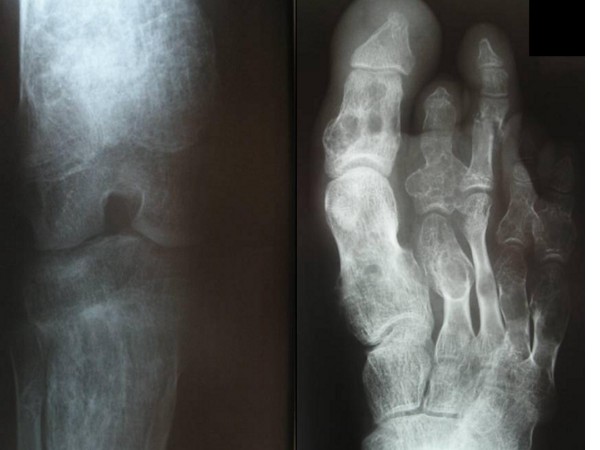
**Anteroposterior lower limb radiograph of the distal femoral, proximal tibial showed enlarged metaphysis near the knees associated with striae of fibro-chondromatosis.** The foot radiograph showed macrodactyly and severe deformities associated with dysplastic 2^nd^, 4^th ^and 5^th ^metatarsals respectively. Note transverse fracture (secondary to minimal trauma) over the proximal phalange of the 3^rd ^toe.

**Figure 5:**
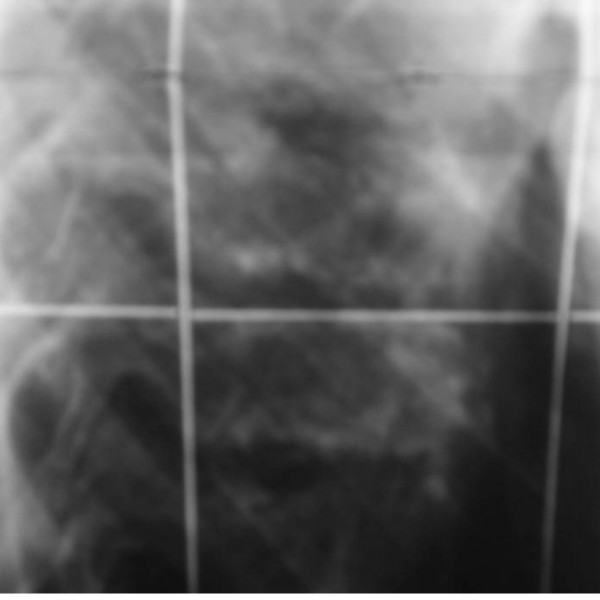
Lateral spine radiograph showed severe lytic changes with extensive irregularities of the anterior/posterior end plates.

At age of 15 years he was found to be severely short with rhizomelic upper limb. His height was 140 cm (-3SD). His head circumference was 58 cm (75^th ^percentile). He had macrocephaly, a course dysmorphic facies (frontal bossing, downslanting palpebral fissures, hypertelorism, long philtrum, broad and large nose and macrostomia). There was generalized ligamentous hyperlaxity associated with genu valgum, valgus ankles and pes planus. Short stature and rhizomelia were evident (fig 1). Radiologically, The anteroposterior hands and forearms radiograph showed irregularly expanded metaphyses and shortened diaphyses were curved over the perimetaphyseal region. Ovoid, cystic and highly radiolucent lesions, elongated parallel to the major axis of the bone, originating near the physis and migrating towards the diaphyses with growth. Shortenings, associated with bone bending causing effectively the development of Madelung's-like deformity (fig 2). Anteroposterior pelvic radiograph showed coxa valga associated with defective modeling of the femoral necks and extensive striae of fibro-chondromatosis (fig 3). Anteroposterior Lower limb- distal femoral, and proximal tibial radiograph showed enlarged metaphysis near the knees associated with striae of fibro-chondromatosis. The foot radiograph showed macrodactyly and severe deformities associated with dysplastic 2^nd^, 4^th ^and 5^th ^metatarsals respectively. Note pathological fracture (secondary to minimal trauma) over the proximal phalange of the 3^rd ^toe (fig 4). Lateral spine radiograph showed severe lytic changes with extensive irregularities of the anterior/posterior end plates (fig 5).

Surgical lengthening of the right femur and the right tibia using an external fixator (Spatial frame), was performed with lengthening of the Achilles tendon of the right side. At the age of 14 years the deformity of the left lower extremity was corrected. His left leg showed a valgus deformity of the distal femur, an external rotation of the left femur of 30° and an anticurvation of the lower leg of 20°. Surgical correction was performed through distal varus osteotomy with a Tomofix plate and a Taylor Spatial frame with osteotomy of the lower leg on the left side and followed by gradual correction, the anatomical alignment was obtained and the external fixation device was removed after 3 months. Renal/abdominal ultrasounds were normal. Echocardiodoppler was normal as well.

He was vigorously investigated. Laboratory findings were completely normal.

## Discussion

Enchondromatosis is a congenital disorder affecting the skeleton in the early stages of development, characterized by persistence of cartilage tissue in the metaphyses and diaphyses. The bone, which is only partially capable of being ossified, has abnormal structure and development, producing changes in morphology and skeletal growth [1-3].

There are different forms of enchondromatosis. The acrofroms (in the hands and feet), monostotic forms (in rays), oligostotic forms, hemimelic forms and generalized forms. The areas most often affected are the tibia and femur (at the knee) and the small tubular bones of the hands and feet. Less commonly involved bones are the fibulae (distally) and the radius and ulna. Involvement of the scapula, ribs and pelvis is uncommon. Involvement of the vertebrae is exceptional [5,6].

Spranger et al., [5,7] called Ollier disease and Maffucci syndrome types I and II enchondromatosis, respectively; metachondromatosis, type III; and spondyloenchondrodysplasia, type IV. Halal and Azouz added 3 tentative categories to the 6 in the classification of Spranger et al., [8].

In Ollier Disease or Multiple Enchondromatosis type I enchondromatosis [5] there is multiple enchondromas of the flat and long bones, distributed unevenly, in various phases of evolution, with exception of the cranium and vertberae. Ollier disase can be present at birth, but it may not become apparent until early childhood. Enchondromas involving long bone are common, leading to progressive skeletal deformities and pathologic fractures with. It occurs in all races with no sex predominance. The diagnosis of Ollier disease is based on clinical and conventional radiological evaluations. Histological analysis has a limited role and is mainly used if malignancy is suspected. Additional investigations, such as scintigraphy, ultrasound, and magnetic resonance imaging are not useful for establishing the diagnosis. In Maffuci syndrome type II enchondromatosis [5], the overall radiographic features are reminiscent to Ollier disease, but with multiple skin haemangiomas. Metachondromatosis is type III, which is characterised by enchondromas, combined with exostosis. Spondyloenchondrodysplasia (type IV) is considered as the modest enchondromatosis of the long bones, unevenly distributed, with severe platyspondyly with mild or no involvement of hands and feet. Enchondromatosis with irregular vertebrae (type V) is characterised by multiple enchondromas of the flat and long bones, with general dysplasia and irregular vertebral bodies, with mild or no involvement of hand and feet. Type VI is general enchondromatosis which is characterised by wide spread of enchondromatosis with severe involvement of the hands and feet with skull deformity. Type VII is generalised enchondromatosis with irregular vertebral lesions and moderate involvement of hands and feet. Type VIII is generalised enchondromatosis with mucopolysaccharoidoses. Type IX enchondromatosis is characterised by concave vertebral bodies [5-11]. None, of the above mentioned entities seem absolutely compatible with our present patient.

Vertebral involvement in multiple enchondromatosis is very rare. Enchondromatosis- vertebral involvement also referred to as the micromelic type of spondylo-meta-epiphyseal dysplasia. Halal and Azouz [8] reported the case of a boy who had platyspondyly and metaphyseal manifestations of enchondromatosis with severe involvement of the hands and feet compatible with generalized enchondromatosis, or Spranger type VI enchondromatosis. The father was short of stature and had only moderate platyspondyly. Both the father and the son had consanguineous parents. They suggested that platyspondyly may be: (1) a manifestation of the carrier state for an autosomal recessive trait; (2) a minor expression of the same autosomal recessive trait in an affected individual since the father's parents were also consanguineous and some of his sibs were reported to have prominent joints; or (3) less likely, variable expression of an autosomal dominant trait in the father and son.

## Conclusion

Distinctive features in our patient include macrocephaly, hypertelorism, downslanting palpebral fissures, long philtrum and a large broad nose. Shortness of stature, short trunk, and rhizomelia were evident. Further noteworthy features were the acrofrom upper limbs with mixed appearance of bilateral and symmetrical radiolucency, cysts and striae of fibro-chondromatosis type, widespread in the forearm bones and the small tubular bones of the hands with severe deformity and the development of Madelung's-like deformity. Lower limbs-femoral, tibial, and fibular dysplasias with enlarged metaphyses near the knees were present. Severe vertebral involvement was additional abnormality.

## Abbreviations

SD: Standard deviation.

## Consent

Written informed consent was obtained from the parents for the purpose of publication of the manuscript and figures of their child. A copy of the written consent is available for review by the editor-in-Chief of this journal.

## Competing interests

The authors declare that they have no competing interests

## Authors' contributions

All of the authors were involved in the clinico-radiographic assessment and finalising the paper. All authors have read and approved the final version of the paper.
